# Effects of low-frequency stimulation of the subthalamic nucleus on movement in Parkinson's disease

**DOI:** 10.1016/j.expneurol.2007.09.007

**Published:** 2008-01

**Authors:** Alexandre Eusebio, Chiung Chu Chen, Chin Song Lu, Shih Tseng Lee, Chon Haw Tsai, Patricia Limousin, Marwan Hariz, Peter Brown

**Affiliations:** a Sobell Department of Motor Neuroscience and Movement Disorders, Institute of Neurology, 8-11 Queen Square, WC1N 3BG London, UK; b Department of Neurology, Chang Gung Memorial Hospital and University, Taipei, Taiwan; c Department of Neurosurgery, Chang Gung Memorial Hospital and University, Taipei, Taiwan; d Department of Neurology, China Medical University Hospital, Taichung, Taiwan; e Unit of Functional Neurosurgery, Institute of Neurology, London, UK

**Keywords:** Synchronization, Basal ganglia, Parkinson's disease, DBS

## Abstract

Excessive synchronization of basal ganglia neural activity at low frequencies is considered a hallmark of Parkinson's disease (PD). However, few studies have unambiguously linked this activity to movement impairment through direct stimulation of basal ganglia targets at low frequency. Furthermore, these studies have varied in their methodology and findings, so it remains unclear whether stimulation at any or all frequencies ≤ 20 Hz impairs movement and if so, whether effects are identical across this broad frequency band. To address these issues, 18 PD patients chronically implanted with deep brain stimulation (DBS) electrodes in both subthalamic nuclei were stimulated bilaterally at 5, 10 and 20 Hz after overnight withdrawal of their medication and the effects of the DBS on a finger tapping task were compared to performance without DBS (0 Hz). Tapping rate decreased at 5 and 20 Hz compared to 0 Hz (by 11.8 ± 4.9%, *p* = 0.022 and 7.4 ± 2.6%, *p* = 0.009, respectively) on those sides with relatively preserved baseline task performance. Moreover, the coefficient of variation of tap intervals increased at 5 and 10 Hz compared to 0 Hz (by 70.4 ± 35.8%, *p* = 0.038 and 81.5 ± 48.2%, *p* = 0.043, respectively). These data suggest that the susceptibility of basal ganglia networks to the effects of excessive synchronization may be elevated across a broad low-frequency band in parkinsonian patients, although the nature of the consequent motor impairment may depend on the precise frequencies at which synchronization occurs.

## Introduction

There is extensive evidence that neuronal activity is abnormally synchronized at low frequencies in Parkinson's disease (PD) and in animal models of parkinsonism (reviewed in Gatev et al., 2006; Hammond et al., 2007; Uhlhaas and Singer, 2006). However, this does not, by itself, prove that pathological synchrony is mechanistically important in parkinsonism. More persuasive evidence would be the impairment of voluntary movement by the artificial synchronization of neural activity in the basal ganglia. Such synchronization is possible by stimulating deep brain electrodes implanted for the treatment of PD at low frequencies, rather than at those frequencies above 100 Hz used for therapeutic benefit. Electrical stimulation of surgical targets like the subthalamic nucleus (STN) simultaneously activates neural elements in the vicinity of the electrode and this synchronous activity is then propagated onwards, as evinced by evoked pallidal (Brown et al., 2004; Hashimoto et al., 2003), cortical (MacKinnon et al., 2005) and muscular activity (Ashby et al., 1999, 2001).

So far there have been several reports of the impairment of movement by stimulation of the STN at frequencies ≤ 20 Hz in patients with PD. Moro et al. (2002) and Chen et al. (2007) studied finger tapping during DBS at 5 Hz and 20 Hz, respectively, and found this to be slowed. However, Timmermann et al. (2004), using the motor United Parkinson's Disease rating scale (UPDRS), failed to confirm a worsening during DBS at 5 and 20 Hz, but did find increased bradykinesia with stimulation at 10 Hz. Another study evaluated tapping performance over several frequencies within the same patients, but only found weak effects that involved relative rather than absolute impairments in motor performance, superimposed upon an overall tendency for movement to improve with increasing stimulation frequency (Fogelson et al., 2005). Accordingly, it is unclear whether stimulation at any or all frequencies ≤ 20 Hz impairs movement and if so, whether effects are identical across this broad frequency band. The issue is an important one, as although spontaneous synchrony tends to occur at frequencies centered around 20 Hz in PD (Hammond et al., 2007), it occurs at rather lower frequencies in the 1-methyl-4-phenyl-1,2,3,6,-tetrahydropyridine (MPTP) primate model of PD (Goldberg et al., 2004; Raz et al., 1996, 2000).

Here we contrast the effects of STN stimulation and thereby extrinsically imposed synchronization at a number of frequencies ≤ 20 Hz, to establish whether all such frequencies impair movement and, if so, whether they impair movement in the same way. To this end we studied performance in a simple finger tapping task, as this is objective and correlates with motor impairment (Giovannoni et al., 1999; Rabey et al., 2002), and considered changes in task execution according to baseline performance (Chen et al., 2006a).

## Materials and methods

### Patients and surgery

Twenty patients participated with informed consent and the permission of the local ethics committees (5 females, mean age 59.5 ± 1.4 years; mean disease duration 13.5 ± 1.0 years). Their clinical details are summarized in Table 1. Fourteen of these patients had also been recorded at least 6 months previously in a different paradigm involving stimulation at 20 Hz, 50 Hz and 130 Hz (Chen et al., 2007). Implantation of bilateral STN DBS electrodes was performed in all subjects for treatment of Parkinson's disease at least 6 months prior to study (mean 34.7 ± 5.9 months). The DBS electrode used was model 3389 (Medtronic Neurological Division, Minneapolis, USA) with four platinum–iridium cylindrical surfaces (1.27 mm diameter and 1.5 mm length) and a centre-to-centre separation of 2 mm. Contact 0 was the most caudal and contact 3 was the most rostral. The intended coordinates at the tip of contact 0 were 10–12 mm from the midline, 0–2 mm behind the midcommissural point and 3–5 mm below the anterior commissural–posterior commissural line. Adjustments to the intended coordinates were made in accordance with the direct visualization of STN in individual stereotactic MRI (Hariz et al., 2003) and, in the patients operated in Taiwan (*n* = 7), the results of microelectrode recordings. Correct placement of the DBS electrodes in the region of the STN was further supported by: [1] effective intra-operative macrostimulation; [2] post-operative T2-weighted MRI compatible with the placement of at least one electrode contact in the STN region; [3] significant improvement in UPDRS motor score during chronic DBS off medication (22.7 ± 3.0) compared to UPDRS off medication with stimulator switched off (52.6 ± 4.8; *p* < 0.00001, paired *t*-test). One patient was excluded due to the absence of significant improvement in UPDRS motor score during chronic DBS and another one due to missing clinical data.

### Protocol

All patients were assessed after overnight withdrawal of antiparkinsonian medication, although the long action of many of the drugs used to treat PD meant that patients may still have been partially treated when assessed. They were studied when the stimulator was switched off and during bilateral STN stimulation at 5 Hz, 10 Hz and 20 Hz. The stimulation types were assessed in pseudo-randomized order across patients, as was the presentation order of trials within a stimulation type. Stimulation contacts, amplitude and pulse duration were the same as utilized for therapeutic high frequency stimulation in each subject (see Table 1). There was no evidence of capsular spread during stimulation, as determined by clinical examination. Patients were not informed of the stimulation type. We did not stimulate one side at a time to avoid possible functional compensation by the non-stimulated side. We waited 20 min after changing between conditions before testing. This is sufficient time to elicit about 75% of DBS effects (Temperli et al., 2003).

### Task

The task was repetitive depression of a keyboard key as fast as possible by rapid alternating flexion and extension of the index finger at the level of the metacarpophalangeal joint (Chen et al., 2006a, 2007). Tapping was performed in two runs of 30 s, separated by ∼ 30-s rest and each hand tested separately (giving four runs per condition). Data from one side were rejected as these were collected contralateral to a previous unilateral pallidotomy (case 18 in Table 1). The number of taps made with the index finger in 30 s was recorded and the run from each pair with the best performance selected for analysis, as this was less likely to be affected by fatigue, or the effects of impaired arousal/concentration.

### Statistics

The results of the tapping task in patients were analyzed according to their baseline performance (e.g. without stimulation). The lower limit of normal baseline performance was obtained by testing ten healthy age matched control subjects (20 sides, 4 males, mean age 57 years, range 52–64 years) using the same tapping task. The mean tapping rate in this control group was 162 taps/30 s. The lower limit of the normal range (e.g. mean − [2 × standard deviation]) in this control group was 127 taps per 30 s. The 35 tapping sides studied in the 18 patients were *accordingly* divided into those with baseline performance within normal limits (*n* = 17; the mean tapping performance across this group, 157 taps/30 s, was still lower than the mean tapping performance in healthy subjects) and those with baseline tapping rates lower than normal limits (*n* = 18; mean tapping performance 58 taps/30 s). The rationale behind this approach was to select those sides (with baseline performance within normal limits) in which any deleterious effects of DBS would not be overshadowed by the beneficial effects of DBS-induced suppression of spontaneous pathological activity or limited by floor effects due to major baseline impairment (Chen et al., 2006a, 2007). Four subjects had sides distributed across the two groups of differing baseline tapping performance. Tapping rates and coefficients of variation were normally distributed (one-sample Kolmogorov–Smirnov tests, *p* > 0.05). Repeated-measures ANOVAs with within-subjects simple contrasts (comparing different frequencies of stimulation to no stimulation) were performed in SPSS (SPSS for Windows version 11, SPSS Inc., Chicago, IL, USA). Mauchly's test was used to determine the sphericity of the data entered in the ANOVAs, and where data were non-spherical Greenhouse–Geisser corrections applied. Means ± standard error of the means are presented in the text.

## Results

**Fig. 1:**
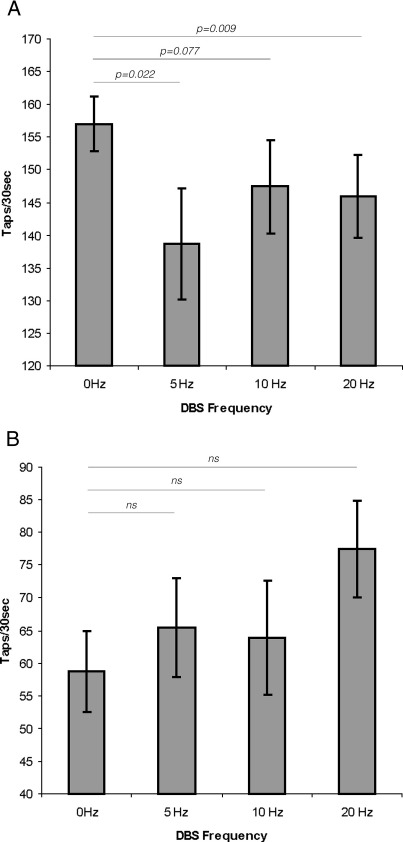
Effects of stimulation frequency on tapping rate off medication. Mean (± SEM) tapping rate off (‘0 Hz’) and on stimulation at 5, 10 and 20 Hz on those sides (*n* = 17) with baseline tapping performance within normal range (tapping off DBS > 127 taps in 30 s) (A) and below normal range (*n* = 18) (B). In those patients with baseline performance within normal limits, tapping rate was significantly slower during stimulation at 5 and 20 Hz than without stimulation and there was a similar trend for the 10-Hz stimulation. On those sides with baseline performance below normal limits no significant differences between the different stimulation frequencies were found.

Low-frequency stimulation had no reliable clinical effect and did not consistently induce tremor, mobile dyskinesia, or dystonic posturing. Four patients experienced dystonic postures during the experiment and one had increased tremor. However, these effects were seen for overlapping stimulation frequencies. We divided the tapping sides into two groups according to whether or not tapping performance off DBS was within normal limits established on 20 sides in 10 healthy age-matched subjects (see Materials and methods). ANOVA of tapping scores with factors FREQUENCY (four levels: 0, 5, 10 and 20 Hz) and BASELINE TAPPING PERFORMANCE (two levels: within normal limits and less than normal limits) demonstrated a within-subjects interaction between FREQUENCY and BASELINE TAPPING PERFORMANCE (*F*_[3,48]_ = 4.224, *p* = 0.01). Data were further analyzed with separate ANOVAs in each baseline tapping performance group. In those patients with baseline tapping performance within normal limits, ANOVA with the factor FREQUENCY confirmed that the latter was a significant main effect (*F*_[3,48]_ = 3.777, *p* = 0.016). Within-subjects contrasts indicated that tapping during 5 and 20 Hz stimulation was worse than during no stimulation (*F*_[1,16]_ = 6.385, *p* = 0.022 and *F*_[1,16]_ =8.793, *p* = 0.009, respectively). The average deterioration in tapping rate during 5 and 20 Hz stimulation compared to no stimulation (0 Hz) in this group was 11.8 ± 4.9% and 7.4 ± 2.6%, respectively (Fig. 1). There was a trend towards a decreased tapping performance at 10 Hz compared to 0 Hz (*F*_[1,16]_ = 3.578, *p* = 0.077). There was no effect of FREQUENCY in patients with baseline tapping performance below normal limits (ANOVA, *F*_[1.9,32.8]_ = 2.202, *p* = 0.128).

**Fig. 2:**
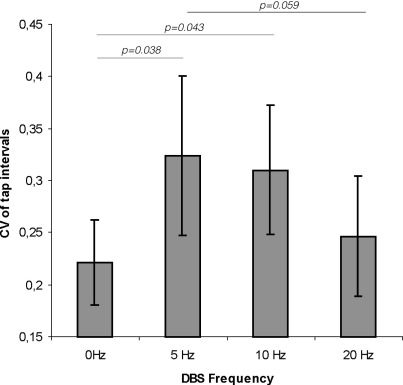
Effects of stimulation frequency on variability of tapping rate off medication. Mean (± SEM) coefficient of variation (CV) of the intervals between taps off (‘0 Hz’) and on stimulation at 5, 10 and 20 Hz in patients with baseline tapping performance within normal range. CV of tap intervals during stimulation at 5 and 10 Hz significantly increased compared to no stimulation.

We also analyzed the variability in tapping as measured by the coefficient of variation (CV) of the time intervals between successive taps on those sides with baseline tapping performance within normal limits. ANOVA with the factor FREQUENCY (four levels: 0, 5, 10 and 20 Hz) revealed a significant effect of FREQUENCY (*F*_[3,48]_ = 3.408, *p* = 0.025). Within-subjects contrasts indicated that the CV increased during 5 and 10 Hz stimulation compared with no stimulation (*F*_[1,16]_ = 5.144, *p* = 0.038 and *F*_[1,16]_ = 4.852, *p* = 0.043, respectively). The average increase of the CV during 5 and 10 Hz stimulation compared to 0 Hz in this group was 70.4 ± 35.8% and 81.5 ± 48.2%, respectively (Fig. 2). There was no difference between the CV at 20 Hz compared to 0 Hz (*F*_[1,16]_ = 0.871, *p* = 0.365). There was, however, a trend for the CV with 5 Hz stimulation to exceed that with 20 Hz stimulation (*t*-test; *p* = 0.059).

## Discussion

We have shown that STN DBS at a variety of low frequencies can slow distal upper limb movements in PD patients with relatively preserved baseline tapping function at the time of study. The effect was present with DBS at 5 Hz and 20 Hz in line with previous studies (Chen et al., 2007; Fogelson et al., 2005; Moro et al., 2002), and there was a trend towards a similar effect with stimulation at 10 Hz (Timmermann et al., 2004). These effects were apparent when tapping sides were separately analyzed according to whether the level of baseline performance was within or outside of normal limits, in line with previous studies suggesting that deleterious effects of DBS are more evident on those sides with relatively preserved baseline performance (Chen et al., 2006a, 2007). The effect was not apparent during stimulation on those sides with impaired baseline performance, either because of confounding, albeit mild, suppressive effects of low-frequency DBS on spontaneous pathological oscillations or because of floor effects (Chen et al., 2007; Fogelson et al., 2005).

In principle, then, the susceptibility of basal ganglia–cortical loops to the effects of excessive synchronization may be elevated across a broad low-frequency band in parkinsonian patients. Accordingly, the relatively different frequency ranges of pathological synchronization in patients and MPTP-treated primates (Hammond et al., 2007) may be more indicative of the resonance properties of basal ganglia networks in the different situations, rather than any fundamental differences in the mechanism of bradykinesia. However, it must be stressed that this is a generalization, and although synchronization at different frequencies may conspire to disturb movement, there may still be subtle differences in the way movement is impaired. This is brought out by the differential effects of low-frequency stimulation on the variation in tapping intervals, evident in differences in the coefficient of variation and hence independent of any differences in tapping rate. Only DBS at 5 and 10 Hz increased temporal variability, whereas DBS at 20 Hz selectively decreased tapping rates without changing tapping variability.

The implication is that basal ganglia networks are involved in processing related to the temporal patterning and regularity of movement and that these circuits may be particularly susceptible to disruption by pathological synchronization at frequencies ≤ 10 Hz. In support of basal ganglia involvement in the temporal patterning of movement, PD patients have increased temporal variability in finger tapping (Giovannoni et al., 1999; Shimoyama et al., 1990), and temporal variability in motor performance is a very early feature of Huntington's disease (Hinton et al., 2007). Indeed, Flowers considered increased variability of movement in both time and space to be one of the core components of motor dysfunction in PD, along with a basic slowness of movement, and a difficulty in initiating and maintaining movement (Sheridan et al., 1987). This variability in motor performance may also relate to the phenomenon of freezing. No overt freezing episodes were observed during tapping in our patients, but an increased variability of stride has been shown in PD patients experiencing freezing of gait independent of frank freezing episodes (Hausdorff et al., 2003).

However, a primary disturbance of temporal patterning is not the only potential interpretation for the increased variability seen during stimulation at 5 Hz and 10 Hz. Tremor was not seen during low-frequency stimulation (except in one patient), in agreement with Timmermann et al. (2004), nor were there any obvious and consistent dyskinesias. Nevertheless, it is possible that synchronization at frequencies ≤ 10 Hz induced subtle hyperkinesias that led to increased temporal variability across taps. A previous case report describes dyskinetic movements induced by STN DBS at 5 Hz (Liu et al., 2002a), and there is increasing evidence that excessive synchronization over 4–10 Hz within basal ganglia circuits may be related to both levodopa-induced dyskinesias in PD (Alonso-Frech et al., 2006) and mobile elements of dystonia (Chen et al., 2006b; Liu et al., 2002b; Silberstein et al., 2003). Relevant in this regard, a recent study demonstrated an increased variability of speech rate in patients treated with l-DOPA and suggested that this effect was related to dyskinesia (De Letter et al., 2006). Variability of swing movement was also observed in the gait of dyskinetic CP children (Abel et al., 2003).

In summary, our results provide further evidence that DBS of the STN over a relatively broad band of low frequencies can impair movement, in line with other more circumstantial evidence of an association between low-frequency synchrony in basal ganglia–cortical loops and altered movement (see recent reviews by Gatev et al., 2006; Uhlhaas and Singer, 2006; Hammond et al., 2007). The present results also raise the important possibility that the detailed profile of motor abnormalities evident in extrapyramidal diseases depends to some extent on the precise frequencies at which pathological synchronization occurs. Indeed, some differences in the details of the effects of pathological synchrony at different frequencies might be anticipated, given the evidence for selective tuning of basal ganglia–cortical sub-circuits (Fogelson et al., 2006).

## Figures and Tables

**Table 1:** Clinical details of patients

Case	Age/Sex	Disease duration (years)	DBS duration (months)	UPDRS III OFF drug − ON/OFF stimulation	Medication	DBS parameters		Pre-op symptoms
1	50/M	8	11	16/31	l-DOPA 500 mg	1-, 3.0 V	60 μs, 185 Hz	Off periods,
5-, 2.5 V	60 μs, 185 Hz	FOG
2	59/M	9	24	8/24	l-DOPA 1800 mg/day Ropinirole 14 mg/day	1-, 3.0 V	60 μs, 130 Hz	Off periods,
6-, 2.5 V	60 μs, 130 Hz	FOG, LID
Amantadine 100 mg/day			
3	58/M	10	22	7/40	l-DOPA 800 mg	1-, 3.6 V	60 μs, 130 Hz	Off periods,
Entacapone 400 mg/day	5-, 3.2 V	60 μs, 130 Hz	FOG
4	55/M	16	94	18/71	l-DOPA 250 mg	2-, 2.5 V	60 μs, 130 Hz	Off periods,
Cabergoline 1.5 mg/day	6-, 2.6 V	60 μs, 130 Hz	FOG
5	63/F	10	39	7/36	l-DOPA 750 mg	0-, 3.0 V	60 μs, 130 Hz	Off periods,
Ropinirole 9 mg/day	4-, 2.4 V	60 μs, 130 Hz	FOG, LID
6	50/F	11	8	37/88	l-DOPA 700 mg	2-, 3.9 V	60 μs, 130 Hz	Off periods,
Ropinirole 9 mg/day	5-, 2.9 V	60 μs, 130 Hz	FOG, LID
7	59/M	11	25	20/46	l-DOPA 600 mg	0-, 2.8 V	60 μs, 130 Hz	Off periods,
Cabergoline 3 mg/day	5-, 3.3 V	60 μs, 130 Hz	FOG
8	64/M	19	30	19/39	l-DOPA 250 mg	2-, 3.0 V	60 μs, 130 Hz	Off periods,
Pergolide 1 mg/day	5-, 3.3 V	60 μs, 130 Hz	FOG
9	68/M	10	10	13/29	l-DOPA 300 mg	1-, 1.8 V	60 μs, 130 Hz	Off periods
Ropinirole 9 mg/day	4-, 2.7 V	60 μs, 130 Hz	
10	51/F	19	22	12/52	l-DOPA 300 mg	2-, 3.3 V	60 μs, 130 Hz	Off periods,
Cabergoline 3 mg/day	5-, 3.0 V	60 μs, 130 Hz	FOG
11	65/M	22	12	6/46	l-DOPA 500 mg	1-, 3.0 V	60 μs, 130 Hz	Off periods,
Pergolide 3 mg/day	5-, 3.5 V	60 μs, 130 Hz	FOG
12	62/F	14	40	35/61	l-DOPA 400 mg	3-, 3.0 V	60 μs, 130 Hz	Off periods,
Entacapone 400 mg/day	7-, 3.0 V	60 μs, 130 Hz	Tremor, FOG, LID
13	67/M	16	80	38/69	l-DOPA 300 mg	L: 2-, 3.3 V	60 μs, 145 Hz	Off periods,
Ropinirole 8 mg/day	R: 2-, 3.0 V	60 μs, 130 Hz (Itrel 2)	Tremor
14	60/M	13	30	24/46	l-DOPA 200 mg	2-, 3.3 V	60 μs, 130 Hz	Off periods,
Pergolide 0.5 mg/day	6-, 3.0 V	60 μs, 130 Hz	LID
Amantadine 300 mg/day			
15	55/M	10	31	33/44	l-DOPA 1000 mg	2-, 2.6 V	60 μs, 140 Hz	Off periods,
Amantadine 100 mg/day	6-, 2.6 V	60 μs, 140 Hz	FOG
Biperiden 2 mg/day			
16	68/M	15	35	40/70	l-DOPA 450 mg	1-, 2.8 V	60 μs, 130 Hz	Off periods,
Amantadine 150 mg/day	6–7+, 3.6 V	60 μs, 130 Hz	DIH
17	59/M	10	33	33/57	l-DOPA 750 mg	1-, 3.5 V	60 μs, 140 Hz	Off periods,
Entacapone 800 mg/day	5-, 3.5 V	60 μs, 140 Hz	Tremor
18^a^	58/F	20	78	42/98	l-DOPA 600 mg	1-, 3.2 V	60 μs, 180 Hz	Off periods,
Bepireden 150 mg/day	1-, 3.0 V	60 μs, 180 Hz	Tremor

Electrode contacts are indicated as convention for Kinetra stimulators (0–3: Left STN; 4–7: Right STN) except for Patient no. 13 (R: Right; L: Left).

a Patient no. 18 was only recorded during Right-hand tapping task due to a previous Right pallidotomy. (FOG: freezing of gait; LID: l-DOPA-induced dyskinesias; DIH: drug-induced hallucinations.)
